# Automatic Roof Plane Detection and Analysis in Airborne Lidar Point Clouds for Solar Potential Assessment

**DOI:** 10.3390/s90705241

**Published:** 2009-07-02

**Authors:** Andreas Jochem, Bernhard Höfle, Martin Rutzinger, Norbert Pfeifer

**Affiliations:** 1 University of Innsbruck, Department of Geography, 6020 Innsbruck, Austria; 2 Vienna University of Technology, Institute of Photogrammetry and Remote Sensing, 1040 Vienna, Austria; E-Mails: bh@ipf.tuwien.ac.at (B.H.); np@ipf.tuwien.ac.at (N.P.); 3 International Institute for Geo-Information Science and Earth Observation, 7500 Enschede, The Netherlands; E-Mail: rutzinger@itc.nl (M.R.)

**Keywords:** airborne LiDAR, 3D point cloud, roof plane detection, classification, segmentation, solar radiation, clear sky index

## Abstract

A relative height threshold is defined to separate potential roof points from the point cloud, followed by a segmentation of these points into homogeneous areas fulfilling the defined constraints of roof planes. The normal vector of each laser point is an excellent feature to decompose the point cloud into segments describing planar patches. An object-based error assessment is performed to determine the accuracy of the presented classification. It results in 94.4% completeness and 88.4% correctness. Once all roof planes are detected in the 3D point cloud, solar potential analysis is performed for each point. Shadowing effects of nearby objects are taken into account by calculating the horizon of each point within the point cloud. Effects of cloud cover are also considered by using data from a nearby meteorological station. As a result the annual sum of the direct and diffuse radiation for each roof plane is derived. The presented method uses the full 3D information for both feature extraction and solar potential analysis, which offers a number of new applications in fields where natural processes are influenced by the incoming solar radiation (e.g., evapotranspiration, distribution of permafrost). The presented method detected fully automatically a subset of 809 out of 1,071 roof planes where the arithmetic mean of the annual incoming solar radiation is more than 700 kWh/m^2^.

## 1. Introduction

In recent years there has been an increasing demand for detailed 3D (three dimensional, expressed e.g., by an xyz coordinate triple) building descriptions from Airborne Laser Scanning (ALS) also referred to as airborne LiDAR data. Adding the third dimension in comparison to 2D, planimetric ground plans allow analysing building heights and their variation, roof shapes and orientation, and visibility studies, in order to name but only a few applications. Particularly, the detection of roof planes can be used in several applications, such as 3D building modeling [1], snow load capacity modeling [2] or selecting suitable areas for the installation of solar panels [3, 4]. The latter is the main focus of this paper, because there is a rising interest in finding suitable roof areas for installation of solar systems for cost effective sustainable energy production [4].

Geometrical information of urban areas of interest can be obtained by using an ALS system, which consists of three main components: (i) a Global Positioning System (GPS), (ii) an Inertial Measurement Unit (IMU) and (iii), a Laser Scanner Unit. While the GPS receiver is used to record the aircraft position, the IMU measures the angular attitude of the aircraft (roll, pitch and yaw or heading). The Laser Scanner Unit transmits pulses of light toward the surface of interest and records both the travel time of the laser beam and the energy which is reflected by the surface [5]. Depending on the Laser Scanner Unit, multiple returns can be detected for a single transmitted pulse. Typically, a part of the emitted pulse is reflected by a tree canopy, whereas gaps on the foliage allow parts of the energy to be reflected further down, e.g., on street level. By taking all flight parameters into account, i.e., measurements by the GPS/IMU systems and the travel time of the laser beam, the target location can be determined with high accuracy in a suited georeferenced coordinate system [6]. Various error factors influence the accuracy of the derived 3D coordinates [7–9]. The 3D information obtained from the Laser Scanner Unit is stored as a point cloud. A point cloud is an unorganized set of 3D points (xyz-triples). The points are distributed irregularly inside surfaces, but typically not found at specific corners or edges. Detailed information on ALS systems can be found e.g., in [10, 11].

A Digital Surface Model (DSM) can be derived from ALS data and describes the Earths surface, including all the objects on the ground. It also contains measurements of buildings, which can be extracted to calculate the solar radiation of an area of interest. This process is however accompanied by two main disadvantages. First, the aggregation of the 3D point cloud to a 2D raster cell often results in a loss of information, i.e., the third dimension and the resolution [e.g., 12, 13]. Secondly, roof planes are not separated from other objects like vegetation and terrain in such models [1]. To maintain the maximum achievable accuracy and hence to perform reliable solar potential computation, it is proposed to perform the process of detection of roof planes and the calculation of attributes like aspect and slope of each roof face directly within the original 3D point cloud. Thus, no down-sampling or initial interpolation is performed. Furthermore, shadows of nearby objects (e.g., vegetation, buildings) are considered by calculating the horizon of each point within the point cloud. This technique avoids the use of a DSM representing objects inadequately due to the interpolation process of the 3D point cloud. Cloud cover effects are respected by using data from a nearby meteorological ground station.

In this contribution we are presenting a new methodology for solar potential assessment of roof planes using the full 3D information of the point cloud. The presented algorithms were fully embedded in a Geographical Information System (GIS) [14], which allows the use of its vector data model and its topological processing tools.

The paper is structured as follows. In Section 2, previous work on building detection, building extraction and solar potential analysis are presented. In Section 3, the methodology and the error assessment of the laser point segmentation are explained, followed by a detailed description of the solar potential analysis in Section 4. The results of the proposed method are described and discussed critically in Section 5. A conclusion is given in Section 6. In this paper the expression solar panel describes two types of devices: (i) photovoltaic devices that convert energy from the sun into electricity and (ii) solar thermal collectors, which use the energy of the sun to heat water.

## 2. Related Work

In this section previous studies concerning building detection, building modeling and solar potential analysis are presented.

### 2.1. Building detection

The process of building detection in ALS data can be performed either on the 3D point cloud or on the resampled 2.5D grid data. The term 2.5D refers to a model that is embedded in 3D-space, but is not able to represent all 3D shapes (e.g., a cave or an overhang). This limitation is given, because for each planimetric (xy) position, only one height (z) is admissible. Using such a model for building detection is less time consuming and is applied in many cases. The 3D point cloud is simplified and aggregated to 2.5D raster cells. Buildings are detected after the generation of a normalized Digital Surface Model (nDSM) by subtraction of the Digital Terrain Model (DTM) from the DSM. The most common features to separate buildings from other objects such as vegetation are roughness, as defined by local height variations, curvature and height differences.

Matikainen *et al*. [15] segment the DSM into homogeneous areas using a region based segmentation, which is based on bottom-up region merging and a local optimization process. The segments are classified as “building”, “tree” and “ground surface”. The classification process is based on the height differences between DSM and DTM, the textural characteristics of the DSM, the intensity image and the shape of the segments.

Forlani and Nardinocchi [16] detect buildings in gridded ALS data by first removing terrain pixels by smooth interpolation and then applying a region growing algorithm to group elevated regions. Classification of the pixels in each region as roof slopes, ridges and building outlines is used to extract the roofs.

Rottensteiner *et al*. [17] present an algorithm for roof line delineation from LiDAR data, which aims at achieving building models at a higher level of accuracy. The algorithm is performed by a segmentation based on local homogeneity of surface normal vectors of a digital surface model.

Some authors developed methods and algorithms to perform building detection directly in the 3D point cloud. Dorninger and Pfeifer [1] propose a comprehensive approach for automated determination of 3D city models from ALS point clouds. The composition of a set of planar faces of buildings can be properly modeled. A 3D segmentation algorithm, which is based on the assumption that points belonging to the same planar region have similar local regression planes, is applied to detect planar faces in a point cloud. This step is followed by a projection of the detected points on the horizontal plane and a regularization algorithm, which derives the building outlines.

Rutzinger *et al*. [12] combine the object-based image analysis approach (OBIA) and the object-based point cloud analysis approach (OBPA) and work partly in 2.5D (raster) and 3D (point cloud). They detect building outlines in the raster domain followed by a 3D roof facet delineation and classification in the point cloud.

Kaartinen *et al*. [18] compare the performance of photogrammetric, laser scanning based and hybrid methods in building extraction within an EuroSDR test. They focus on the determination of building outlines, lengths and roof inclination and confirm that laser scanning is more suitable for deriving building heights, extracting planar roof faces and ridges of the roofs. Photogrammetry and aerial images lead to better results in building outline and length determination.

### 2.2. Automatic building reconstruction

Two methodological approaches for automatic building reconstruction are predominant, the (i) modeldriven and the (ii) data-driven approach. The first type searches the most appropriate model among basic building shapes contained in a model library and is generally applied for low point densities. This method leads to roof shapes that are always topologically correct. Its disadvantage is that complex shapes can not be modeled in a proper way because they are not included in a library of models. The second type attempts to reconstruct a building from building parts found by segmentation algorithms [19] and is appropriate for high point densities. Neighboring roof segments have to be identified and intersected with each other. Small roof elements may cause problems because they may not be detected by the segmentation process [1].

Maas and Vosselman [20] present a method for the automatic derivation of building models from laser altimetry data, which is based on the analysis of invariant moments of point clouds. The moments used in the analysis are e.g. the second centralized moment, corresponding in 1D to the variance and in 2D to the covariance matrix. The moments and their relative values provide information on the roof shape, e.g., orientation, independent from the location within the coordinate frame.

An application using the data-driven approach is proposed by Vosselman and Dijkman [21]. A three-dimensional version of the Hough-transform algorithm is used to detect planar faces within the unstructured point cloud. Reconstruction of the buildings is performed by using available ground plans of the buildings.

Further applications using either the model-driven or the data-driven approach for automatic building reconstruction can be found in Tarsha-Kurdi *et al*. [13] and Tarsha-Kurdi *et al*. [19].

Oude Elberink [9] focuses on problems related to the reconstruction of building parts using either the data driven or the model driven approach. Examples are shown of ALS data with an average point density of 25 points per square meter.

### 2.3. Solar potential analysis

The development of algorithms that automatically classify and segment LiDAR point cloud data enables the detection of suitable areas for the placement of solar cells at unprecedented level of detail. The methods have been developing from a manual to an automatic selection of appropriate areas.

Wittman *et al*. [22] measure the roofs with respect to aspect, inclination, and size for an area of 0.9 km × 1.2 km (Vienna, Austria) by means of photogrammetry and determine areas, suitable for the installation of solar panels.

Vögtle *et al*. [3] use ALS data to select suitable areas for the installation of solar panels automatically. The extraction of the roof planes and the determination of the required features such as size, aspect and slope are performed on a DSM. By means of building footprints these attributes are assigned to individual buildings. The selection process is done within a GIS database management system.

Kassner *et al*. [4] mask ALS data by the outlines of the buildings in order to obtain information about the roof of the building. A raster interpolation of the remaining points is performed to analyze the roofs according to aspect, slope and shaded areas.

## 3. Methods

### 3.1. Test site and datasets

The test site is located in an urban settlement in the city of Feldkirch (Vorarlberg/Austria) and covers an area of approximately 1 km × 1 km. Besides single houses and block buildings with mainly ridged roofs, the test site contains small structures such as cars, fences and vegetation of different geometry and types. In many cases the vegetation is found very close to buildings. This can lead to challenges separating buildings from vegetation, particularly when branches of nearby trees cover parts of a roof.

The data used for the development of the presented algorithm were provided by the Federal State of Vorarlberg. Laser scanning data are available for the whole area of the state and were acquired in 2004 by a Leica ALS-50 scanner with a wavelength of 1064 nm, a pulse repetition frequency of 57 kHz, a maximum swath width of 75° and maximum scan rates of 75 Hz. The average point density within the area of Feldkirch is 17 points/m^2^ [23]. The official DTM of Vorarlberg with 1 m resolution was available for this study. It was generated in the framework of the country-wide ALS project of the Federal State in 2004 using the method of robust interpolation [24]. An orthophoto covering the whole test site was created in 2006 and was also available for the research. Additionally, a shaded relief map based on a 1 m DSM was created for the whole test site.

### 3.2. Workflow

**Figure 1.**
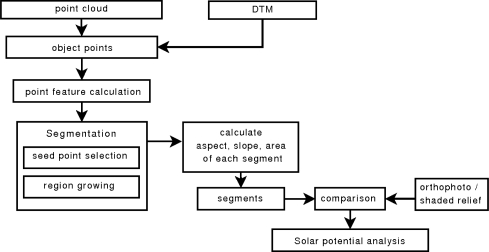
Workflow for roof plane detection in 3D point cloud.

For building detection from ALS data, two successive steps are performed. First, the terrain points are separated from the object points [13], and secondly the objects of interest are detected within the off-terrain points. By subtracting the terrain from the absolute point heights and removing points below a defined relative height threshold, the influence of the terrain and low objects such as small vegetation, cars, fences etc. are eliminated. The calculation of point features such as its normal vector and surface roughness, followed by a seed point selection and region growing process are applied to detect the roof planes in the 3D point cloud. To determine the solar radiation of each roof segment, its inclination, aspect and area are calculated. Error assessment based on orthophotos and a shaded relief is performed to estimate the accuracy of the roof detection process. The proposed workflow is shown in Figure 1.

### 3.3. Selection of object points

The presented algorithm focuses on roof planes only. Therefore, points having a height of more than 2 m above the terrain are selected for the classification and segmentation process. To remove those points that are not of further interest (terrain points, points on cars, etc.), the relative height value is derived by subtracting from each laser point elevation the elevation of an underlying DTM. After applying the threshold on relative height (> 2 m), the points with their original elevations are used for further processing. Using the relative height value of each laser point would lead to deformations or change of orientation of roof planes where the underlying DTM is not strictly flat such as in hilly or sloped terrain.

### 3.4. Feature calculation

In contrast to other high objects detected by an ALS, like vegetation, roofs are composed of one or more planar parts, i.e. roof planes, dependent on the roof type. Thus, points belonging to the same planar region must have similar normal vectors, which can be estimated by fitting an orthogonal regression plane to each point and its *k* nearest neighbors [12]. The surface roughness, defined as the standard deviation of the orthogonal fitting residuals [25], is used as an additional feature to verify local planarity of a point.

### 3.5. Seed point selection and region growing

The lower its local roughness, the more likely the chance that a point lies on a planar face. Hence, all points are ordered by ascending roughness. The points having a roughness value below a defined threshold are potential seed points for the region growing process. The seed point’s *k* nearest neighbors are evaluated by: (i) similarity of normal vectors and (ii) 3D distance between seed point and neighbor. If a neighbor fulfills both criteria, it is accepted as belonging to the segment and is used as a next seed point.

Similarity of normal vectors is fulfilled if the angle between the normal vector of the seed point and the normal vector of the neighbor is within a predefined threshold. A maximum distance between the seed point and its neighbors is used to check the second criterion.

Once the current segment reaches a predefined number of points an orthogonal regression plane is fitted to the segment and its normal vector is taken as reference vector to verify the similarity to the normal vectors of the candidate points. This step is repeated as soon as a new point becomes part of the current segment. A segment grows until it reaches the ridge or the edge of a roof. Points being part of a segment are removed from the available points and the algorithm continues until all potential seed points are used. Defining a minimum number of points per segment helps to remove small segments that are not of further interest, e.g., chimneys.

### 3.6. Calculation of slope and aspect

Slope (*γ*) and aspect of the detected roof planes are of fundamental importance to perform solar potential analysis. The slope is determined by calculating the angle between the normal vector of the fitted plane (Section 3.5.) and the z-axis.

The aspect of each roof plane is determined by the angle between the projected normal vector on the horizontal plane (xy-plane) and the geographic north direction. The latter is typically the y-axis, but because of cartographic projection small deviations may occur.

### 3.7. Area of segments

The area of each segment is required to calculate its potentially available solar radiation. According to Höfle *et al*. [25] and Da [26] two dimensional alpha shapes [27] can be used to derive the outline of a dense unorganized set of data points in 2D space. An alpha shape of a given finite point set *S*, expresses the intuitive notion of the “shape” of *S* as a polytope that is determined by *S* and a real parameter *α*, whereas *α* controls the level of detail reflected by the polytope [28].

**Figure 2.**
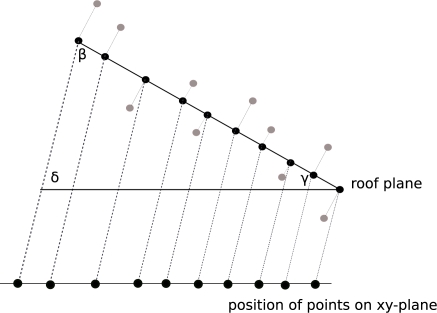
The projection of each point lying on a roof plane on the xy-plane. The dashed line is determined by the inclination angle *γ* of each roof segment. For simplicity the figure is shown in 2D profile.

**Figure 3.**
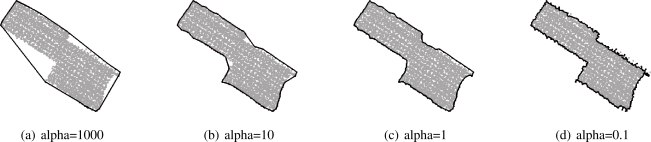
Segment boundary derived by alpha shapes for different values of *α*. For large values of *α* the convex hull is obtained (a), whereas small values lead to outlines with artifacts caused by the random point distribution (d).

To determine the outline of a roof segment, all points representing a roof plane in 3D space are projected on the xy-plane by maintaining the real area of each segment as shown in Figure 2. Before projecting the points on the xy-plane each point is orthogonally projected on the plane of the current roof segment. The slope *γ* of each segment determines the equal angles *β* and *δ* (Figure 2) and consequently the direction, which is needed to project each point on the xy-plane. Once the points are projected two dimensional alpha shapes are used to derive the area of each roof plane. The alpha value *α* is the determining factor for the resulting shape of the boundary of the segment. As one can see in Figure 3 the segment boundary varies as a function of *α*. A large alpha value (*α* → ∞) results in a shape representing the convex hull. A very small alpha value (*α* → 0) degenerates the alpha shape to the point-set [26]. The average point distance is a good estimate to find an optimal alpha value that produces the exterior boundary of the current segment.

### 3.8. Error assessment

This paper focuses on the solar potential analysis of roof planes. Therefore, it is important to detect as many roof planes as possible. Due to lack of terrestrially measured data of roof planes and the occurrence of shifts in the available orthophotos, the accuracy of the position of the detected roof planes can not be determined as adequately as in e.g. Kaartinen *et al*. [18]. Hence, an object-based error assessment is performed to check completeness of the extracted roof planes. Each roof plane within the selected area is labeled with a reference point in its center on the basis of orthophotos and a shaded relief. The shaded relief calculated from the DSM is additionally used because the ALS data and the orthophotos were acquired in different years and might differ in some regions. Small dormers are not considered because during the segmentation process, small segments not having a minimum number of points are removed (Section 3.5.). They are not suitable for the installation of solar panels. The digitized points are compared to the derived polygons (Section 3.7.) by performing a point in polygon test. The accuracy of the segmentation is expressed by completeness and correctness.

## 4. Solar Potential Analysis

### 4.1. Theory

According to Šúri and Hofierka [29], three factors determine the interaction of the solar radiation with the Earth’s atmosphere and surface: (i) The geometry of the Earth: its rotation and revolution around the sun determines the available extraterrestrial radiation based on solar position above horizon. (ii) The topography of the terrain, i.e., the slope, the aspect and shadowing effects of neighboring terrain features, modifies the distribution of the radiation input to the earth surface. (iii) The attenuation of the atmosphere caused by gases, solid and liquid particles and clouds.

The first two factors can be modeled at a high level of accuracy using astronomic formulas. Due to the dynamic nature of the atmosphere and its complex interactions, modeling the atmospheric attenuation is still a challenging task and reaches only a certain level of accuracy.

The air mass and optical thickness, which influence the attenuation by gas can be calculated at a good level of accuracy using formulas proposed by Kasten and Young [30]. The attenuation by solid and liquid particles can be described by the Linke turbidity factor, which indicates the optical density of hazy and humid atmosphere in relation to a clean and dry atmosphere. Values for the Linke turbidity factor differ between geographical location and season and can be taken from literature [31]. The calculation of the atmospheric attenuation depends on a number of variables, such as position and number of layers of clouds, their optical properties and their instantaneous thickness. Detailed descriptions can be found in Šúri and Hofierka [29], Kasten and Young [30], Hofierka and Šúri [32], Kasten and Czeplak [33], and Kasten [34].

This paper proposes a model, which estimates the global solar radiation of a point of interest under clear sky as well as under cloud covered conditions. The global solar radiation is calculated by the sum of the direct and the diffuse radiation. The direct radiation is the part of the radiation which reaches the surface directly without being reflected or scattered by the atmosphere. The diffuse radiation is scattered radiation that reaches the surface. Formulas, estimating the direct and the diffuse component are taken from [32]. A short overview is given in Appendix 7.. Sunrise and sunset times, the position of the sun and its incidence angle on the surface are computed using the SOLPOS Code developed by the National Renewable Energy Laboratory [35].

Cloud cover effects are considered by using data from a nearby meteorological station and calculating the clear sky index *k_c_*, defined in Equation 1. On horizontal surfaces it is defined as the ratio of the global radiation under overcast conditions *G_h_* and clear sky conditions *G_hc_* [29].

(1) $$k_{c}=G_{h}/G_{\mathrm{hc}}$$

An inclined surface has a different ratio of direct and diffuse radiation than a horizontal surface. Therefore the direct and the diffuse component should be treated separately and the clear sky index has to be determined for each of the two components. In this case the meteorological stations have to measure both the diffuse and the direct component of the global radiation. In our approach the clear sky index used for horizontal planes is also used for inclined roof facets due to lack of data from meteorological stations. Once the clear sky index is determined, the direct and the diffuse component under overcast conditions on horizontal and inclined surfaces can be calculated [29, 32].

To avoid a loss of information, the extracted points (Section 3.) are not interpolated to a raster. The solar potential analysis is performed at a sub segment level. For this purpose each segment is represented by a number of uniformly distributed points (Section 4.2.). For each of these points the solar potential is determined.

### 4.2. Uniform distribution of points

It cannot be generally assumed that the distribution of the recorded laser points is uniform. Due to overlapping flight strips and changing airplane attitude (mainly pitch) the distance between points as well as the point density vary, even within one roof plane. Therefore, we propose a discretization of the derived roof planes to avoid an over representation of a certain part of the roof when calculating the arithmetic mean of the incoming solar radiation per roof segment. An algorithm was developed, which places uniformly distributed points in 3D space within the boundaries of each segment by adjusting to a suited grid. In the following these points are called *uni-points*. A spacing of 0.3 m is used between the segment points. This is applied only to that face, for which the incidence radiation is currently computed.

### 4.3. Shadowing effects

**Figure 4.**
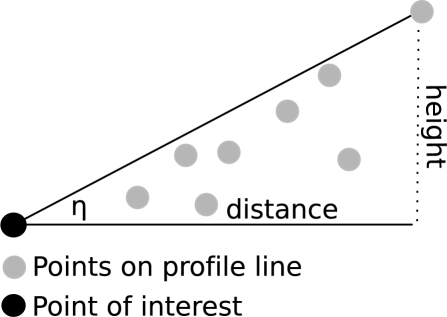
The horizon is calculated for each point for each solar azimuth angle that is used to compute the global solar radiation. The angle *η* determines the minimum solar elevation angle such that an *uni-point* is not in the shadow of a nearby object. For simplicity it is illustrated in 2D.

In this paper, shadowing effects of the surrounding terrain are not respected directly. They are included in the clear sky index (Equation 1). The global radiation under clear sky conditions on a horizontal surface (very close to the meteorological ground station) is modeled by considering the shadows of a DTM. This procedure was chosen because on clear sky days the meteorological ground station is also affected by shadowing effects of the surrounding terrain. Values under overcast conditions are represented by 30-years measurements of the global radiation of a nearby meteorological ground station. Shadows of neighboring objects are variable for each roof facet and are considered by calculating the horizon of each individual point within the original point cloud. Therefore, a line from the point of interest to a point lying in a defined distance in annual minimum solar azimuth angle direction is created. All points within a defined distance to the line are taken to check the horizon of the point of interest in the current direction as illustrated in Figure 4.

The ratio between distance and difference in height determines the angle *η*. The maximum angle within the profile line is equal to the minimum solar elevation angle that is needed to have a line of sight between the point of interest and the sun i.e. the point is not within a shaded area. Once the angle *η* is determined, the line is rotated clockwise in defined degree increments and the horizon is calculated for the current direction. This step is repeated until the line reaches the annual maximum solar azimuth angle.

Thus, one gets the minimum solar elevation angle for each solar azimuth angle. Points having no neighbors within a defined distance because of reflections from small objects e.g. birds, lanterns etc. are removed from the profile line and are not considered for computing the horizon. If there are power lines within the area, methods for linear feature extraction based on eigenvalues could be used to determine and exclude those points [36]. Furthermore, points within the profile line must have a defined minimum distance to the point of interest. This avoids points being very close to the point of interest and differing in elevation (due to noise occurring during measurement) casting a shadow.

**Figure 5.**
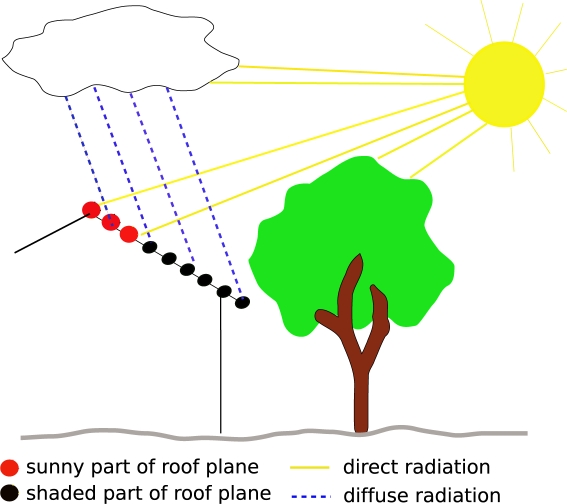
The direct radiation on the roof plane is influenced by a nearby tree. Not all parts of the roof facet receive solar energy. Once a point is in the shade of an object, its direct radiation is set to zero and only the diffuse component is considered.

For each sun position, which is used to compute the global solar radiation, the angle *η* is checked. If a point is within a shaded area its direct radiation is set to zero for the current sun position and only its diffuse component is respected. Hence, partly shaded roof planes are also considered and one can see which part of the roof plane is suitable for the installation of solar panels. Figure 5 illustrates shadowing effects considered in the presented approach.

### 4.4. Implementation

Table 1 shows the algorithm which is applied on the extracted roof segments.

The solar potential analysis is performed for each *uni-point* of a segment, whereas the normal vector of each point is equal to the normal vector of the segment the point is belonging to. This is reasonable because solar panels are planar facets and their surface does not vary either.

The sun position is calculated for each day of the year from sunrise till sunset in one hour steps. Each *uni-point* is treated separately. If the considered *uni-point* is within a shaded area, its direct radiation is set to zero and only the diffuse component is calculated. If the position of the sun is less than one hour before sunset, the global radiation *G_rad_* is given as described in Equation 2:

(2) $$G_{\mathrm{rad}}=G_{\mathrm{rad}}*{\mathrm{rem}}_{\mathrm{sunset}}/60$$

where *rem_sunset_* are the remaining minutes until sunset. As a result of the algorithm, the annual sum of the direct and diffuse radiation on each laser point lying on a roof plane is calculated. In a next step, the arithmetic mean of the solar radiation per segment can be calculated and multiplied with its size (Section 3.7.). Thus, one gets the available solar energy per roof segment.

## 5. Discussion

In this section the results of the roof segmentation process, error assessment and solar potential analysis are presented.

### 5.1. Roof plane detection

**Figure 6.**
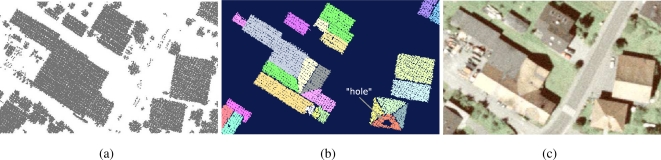
Roof face extraction, a) selected object points showing vegetation, roofs and other objects, b) roof points detected by region growing, c) orthophoto for comparison.

As a result of the region growing process (Section 3.5.) all points covering roof planes are detected, classified by roughness and segmented into homogeneous areas of similar normal vectors. Each segment represents one roof plane and vegetation is removed (Figure 6). Best results are achieved by applying the settings shown in Table 2.

A roughness threshold of 0.35 meters removed points on non-planar objects like e.g. vegetation from the potential seed points list. A maximum distance value of 0.5 m from the current seed point to the next potential seed point is chosen during the region growing process. Furthermore, the angle between the compared normal vectors should be within a threshold of 17 degrees to keep the current segment growing. A neighborhood of 27 nearest neighbors and a minimum segment size of 90 points turned out to be the best parameters for the segmentation.

**Figure 7.**
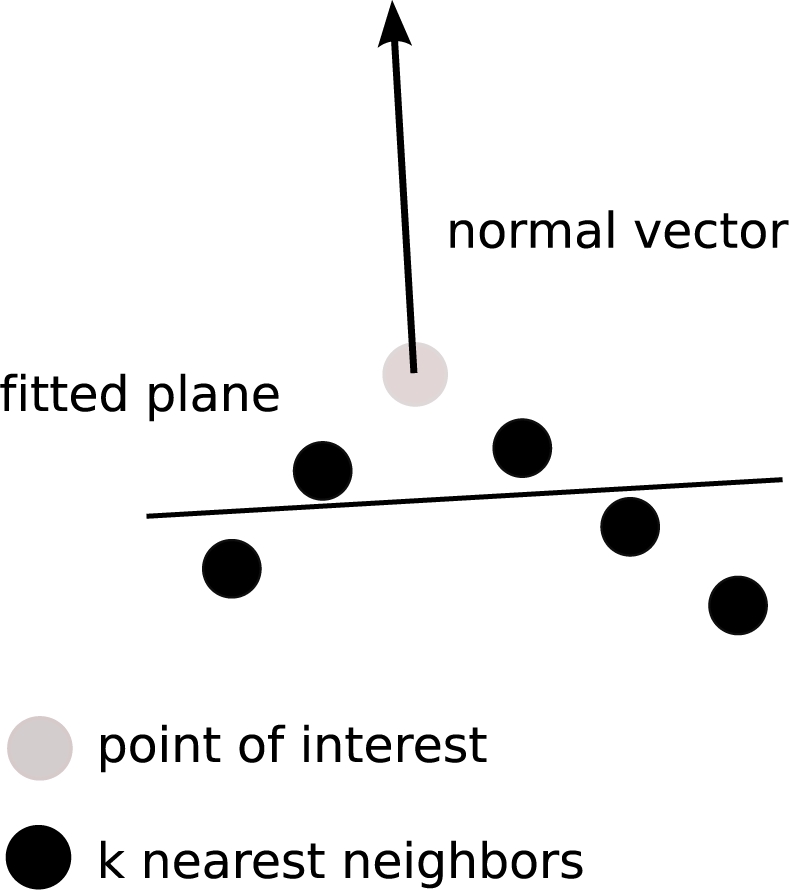
In many cases the normal vector on roof ridges differs beyond a defined angle threshold from those on the roof plane. Therefore, roof ridges often are not considered by the segmentation algorithm.

As one can see in Figure 7(b), roof ridges are not always detected by the segmentation algorithm. Points having a normal vector that differs beyond a defined angle threshold do not become part of the current segment.

As illustrated in Figure 7, the normal vector of points on roof ridges is more or less vertical to the ground. Note that the number *k* of nearest neighbors strongly influences the resultant normal.

Depending on the defined angle threshold and the inclination of the roof facet, the angle between the normal vector of points on roof ridges and the normal vector of the current segment exceeds the threshold in many cases. Another reason for missing roof ridges is the roughness value of each point. It is higher on roof ridges than on other parts of the roof. Using a robust plane fit for feature calculation would improve the results and the segment could grow closer to the ridge. A higher point density would result in the same effect, because the neighborhood (spatially seen) of each point for feature calculation can be reduced. The area of each roof plane as determined by the algorithm can in practice deviate from the actual area that is available for the installation of solar panels. Figure 7(b) shows that the area can be disturbed by “holes”, caused by chimneys, dormers or windows, that were closed by alpha shapes. Therefore not all of the detected area of the roof facet might be suitable for the installation of solar panels. These cases are not considered yet but are planned for future studies.

When branches of nearby vegetation cover parts of a roof facet it may become difficult to distinguish between points on buildings and vegetation points. In some cases it is impossible to detect roof facets below dense vegetation because laser shots did not reach the surface of the roof and thus it is not represented in the point cloud. In other cases vegetation points became part of a roof plane because the segmentation algorithm cannot distinguish between planar faces and vegetation. Either the vegetation has planar characteristics similar to the neighboring roof facet or a high percentage of roof points was used to calculate features (i.e., normal vector, roughness) of a point covering vegetation and influenced the results. Hence the segment grows until none of the *k* nearest neighbors of the seed points fulfills the predefined conditions. A high percentage of detected roof facets including vegetation could be corrected by adjusting the parameters (Table 2) of the segmentation process. Another problem occurred with hedges within the selected area. Due to their density and height above ground (i.e., >2 m) they are also recognized as planar patches and could hardly be removed by adjusting settings.

### 5.2. Error assessment

Within the selected area, 1,003 roof planes were visually identified and labeled by an inspection of the available shaded relief and orthophoto products. The detection procedure results in 1,071 roof areas, whereas 947 of them correspond to the reference dataset. Consequently, 56 were not detected and 124 were not correctly classified. These results lead to a completeness of 94.4% and a correctness of 88.4%.

The applied error assessment includes both the (i) quality of segmentation and (ii) quality of classification. The latter is included in completeness and correctness. The former can be expressed by checking over- and undersegmentation. Oversegmentation occurs if a roof plane labeled with reference point is segmented in more than one segment. Undersegmentation is the case if several labeled roof segments are detected as one segment. Neither of them occurs in our approach.

### 5.3. Solar potential analysis

**Figure 8.**
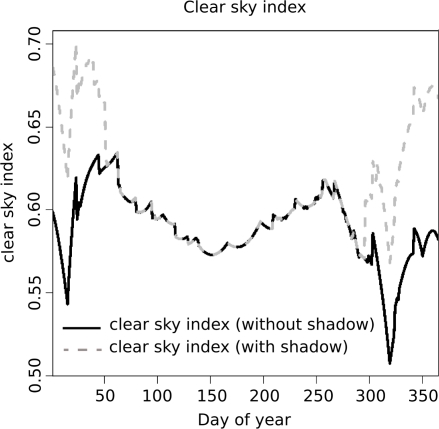
The clear sky index over the course of the year. The dashed grey line shows the clear sky index by respecting the shadows of the DTM. The black line does not include any shadows.

Figure 8 shows the clear sky index over the course of the year, respecting and not respecting shadows of the DTM, which was resampled to a resolution of 10 m.

The closer the clear sky index is to 1.0, the more the modeled and the measured values coincide. The location of the meteorological ground station is strongly influenced by shadows of the terrain during the winter time, when the sun elevation angle is relatively small. From spring till fall these shadows are negligible and the meteorological ground station is only affected by clouds.

**Figure 10.**
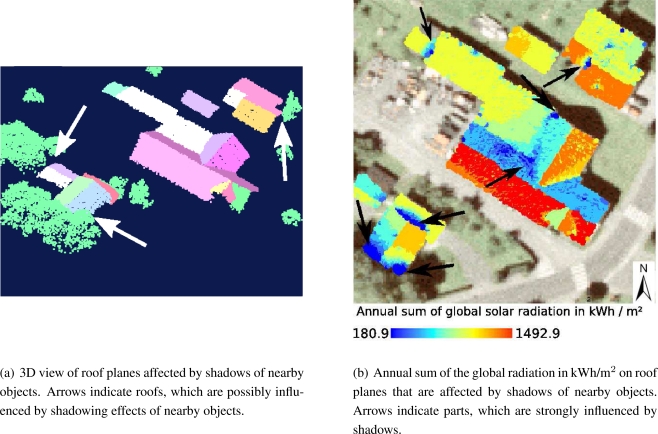
Point cloud view of detected roof planes together with objects possibly casting a shadow (a) and results of solar potential assessment for selected roof planes (b).

Figures 10(a) and 10(b) shows the results of the solar potential analysis for surfaces of different inclination and aspect angles within the test area. Both, the distribution of the global radiation under clear sky and under overcast conditions are shown over the course of the year. Shadowing effects of nearby objects are not respected in this case. They differ between the roofs and are variable for each building.

In Figure 10(b) the clear sky index, which is computed for every single day is used to correct the modeled values shown in Figure 10(a). Figure 10(a) also includes the measured radiation of the nearby meteorological ground station.

This is equal to the line representing a flat roof in Figure 10(b), because the clear sky index is the ratio between the measured values and the values modeled for a flat roof. While a flat roof reaches a maximum global solar radiation value of 8.754 kWh/m^2^/day under clear sky conditions (Figure 10(a)), its maximum value is 5.068 kWh/m^2^/day under real sky conditions (Figure 10(b)). Furthermore, it can be asserted that the inclination angle of the roof and its aspect plays an important role in both cases, under clear sky and under real sky conditions. Particularly during the winter time (when the demand for energy is higher than during summer time), south orientated roof panels gain much more energy than those orientated in other directions. During the summer time flat surfaces receive the most solar energy. This is due to the solar incidence angle, which is measured between surface and sun ray and is the steepest on horizontal surfaces during that time. A further result is shown in Figure 10. The horizon of each point is used to respect shadows of nearby objects i.e vegetation, buildings and the roof itself. Parts of a roof, which are covered by shadows receive less energy than uncovered ones and one can see which part is suitable for the installation of solar panels. By using methods for linear feature extraction to determine and exclude points on e.g. power lines from the 3D horizon, roof ridges are not considered either as an object point casting a shadow.

**Figure 11.**
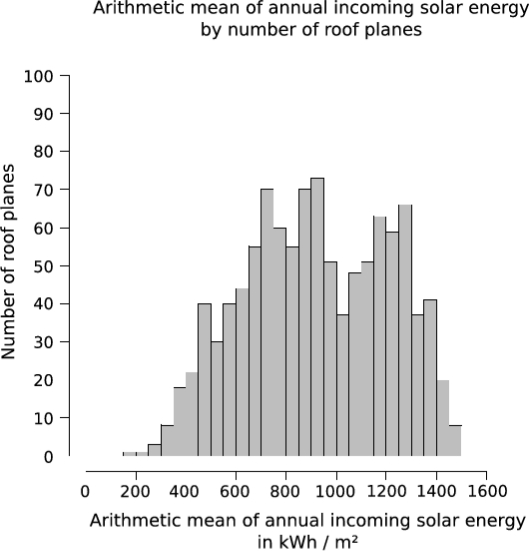
Arithmetic mean of annual incoming solar energy by number of roof planes. 75.5% of detected roof planes receive more than 700 kWh/m^2^ of solar energy.

Having more detailed data from meteorological ground stations and determining the clear sky index for the diffuse and the direct radiation separately can lead to better results, particularly on inclined surfaces. As mentioned above (Section 4.3.) the clear sky index already includes shadows of the terrain but not of the surface. On clear sky days, the meteorological ground station is also affected by the surrounding terrain. If one does not consider these shadows in the clear sky index, this index will be underestimated. Failures in calculation might occur at locations that are affected by shadows of the terrain, which do not influence the meteorological ground station. Another aspect, which can be taken into account is that during winter time the influence of nearby trees on a roof plane is less than during summer time. Transparent shadow values could be introduced and improve the results. Figure 11 show a breakdown of the detected roof planes with respect to their arithmetic mean of annual incoming solar energy, by number of roof planes. A high percentage (75.5%) of detected roof planes receive more than 700 kWh/m^2^ of solar energy. If they are suitable for the installation of solar panels must be clarified by detailed planning.

## 6. Conclusions

In recent years, a lot of algorithms have been developed which calculate the solar potential on basis of 2.5D raster data. However, rasterization of the point cloud is always accompanied with loss of information and thus the full potential of the ALS data concerning accuracy and resolution is not exploited. Particularly, when calculating the solar radiation of roof planes, their aspect, inclination angle and area play an important role. They are the determining factors of the solar potential analysis and have a significant impact on the results.

The presented method detects roof planes in the 3D point cloud with 94.4% completeness and 88.4% correctness and maintains the maximal achievable accuracy. Shadowing effects of nearby objects are considered by computing the horizon of each point within the 3D point cloud. Using a DSM would lead to deviations at roof overhangs, chimneys, dormers etc. due to the rasterization process and thus shadows are not represented properly. Furthermore, single high points e.g. on an antenna, would affect the roof facet like a wall, which is not there. Single high points of the point cloud are not considered as belonging to the horizon in the presented approach, and thus no shadow will be cast. Cloud cover effects are respected by determining the clear sky index, which also includes shadows of the terrain.

This approach allows the detection of suitable roof planes in a fast, accurate and cost effective way. In particular areas which are strongly affected by shadows can be excluded from a potential suitable areas list quickly. Detailed plannings including determination of the optimal inclination angle of solar panels and the available area are required for each building, which seems to be suitable for the installation of such devices.

However, this method cannot only be used to perform solar potential assessment of roof planes. It can be applied to a variety of applications. Many natural processes are directly influenced by the incoming solar energy e.g. permafrost distribution [37], evapotranspiration (sum of evaporation and transpiration) [38] and hence it can be thought about integrating this approach in models simulating these processes.

Nevertheless, many improvements can still be made (Section 5.) and are planned for future studies. The presented approach shows promising results and offers a number of applications.

## Figures and Tables

**Figure 9.**
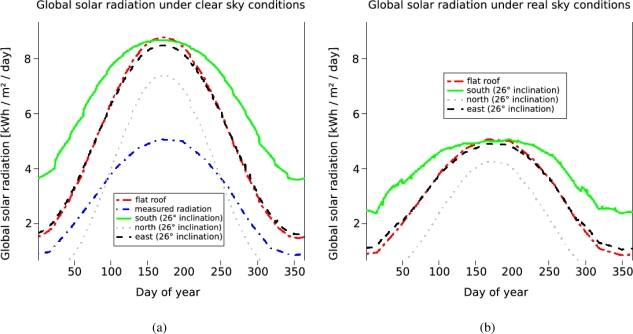
Distribution of the global solar radiation over the course of the year under clear sky (a) and under real sky (b) conditions.

**Table 1.** Pseudo Code for solar potential analysis.

**Input:**	P*_xyz_* (roofs) (*uni-points* representing roof planes including aspect, slope, horizon for each point)
	k*_c_* (clear sky index for each day)
	sunrise, sunset, position of sun (computed from SOLPOS Code)
**Output:**	P*_solrad_* (solar radiation for each point [Whm^−2^])
P*_solrad_* = 0	
**for** i = 1 **to** 365 **do**	
step = 0	
compute hours of sunshine for day i (sunset - sunrise)	
**repeat**	
compute position of sun for sunrise + step	
**for** r = 1 **to** r = amount of P*_xyz_* (roofs) **do**	
**if** sunrise + step <= sunset **then**	
**if**$P_{\mathrm{xyz}}^{r}$ (roofs) is in shadow **then** direct beam is zero	
calculation of global radiation G*_rad_* for $P_{\mathrm{xyz}}^{r}$ (roofs)	
$P_{\mathrm{solrad}}^{r}=P_{\mathrm{solrad}}^{r}+G_{\mathrm{rad}}$	
**else**	
calculation of remaining minutes till sunset (rem*_sunset_*)	
**if**$P_{\mathrm{xyz}}^{r}$ (roofs) is in shadow **then** direct beam is zero	
calculation of global radiation G*_rad_* for $P_{\mathrm{xyz}}^{r}$ (roofs)	
G*_rad_* = G_rad_* (*rem_sunset_*/60)	
$P_{\mathrm{solrad}}^{r}=P_{\mathrm{solrad}}^{r}+G_{\mathrm{rad}}$	
**end**	
step=step+1	
**until** (sunrise + step *>* sunset)	
**end**	

**Table 2.** Applied settings for roof plane detection.

roughness threshold	0.35 m
maximum distance	0.5 m
angle between normal vectors	17°
*k* nearest neighbors	27
minimum segment size	90

## A. Formulas

### A.1. Direct radiation

The direct radiation *B* [Wm^−2^] is calculated as follows:

(3) $$B=G_{0}\;\text{exp}\;\{-0.8662T_{\mathrm{LK}}\;m\delta_{R}\;(m)\}\;\text{sin}\;\delta_{\mathrm{inc}}$$

where:

*G*_0_ is the corrected extraterrestrial irradiance normal to the solar beam [Wm^−2^];
−0.8662*T_LK_* is the corrected air mass 2 LINKE atmospheric turbidity factor;
*m* is the relative optical air mass;
*δ_R_*(*m*) is the Rayleigh optical thickness at air mass m;
*δ_inc_* is the solar incidence angle between the sun ray and the surface.

To consider the varying sun-earth distance across the year the solar constant *I*_0_ [1367Wm^−2^] is corrected by the factor *ɛ*:

(4) $$G_{0}=I_{0}*ɛ$$

where:

(5) $$ɛ=1+0.03344*\cos(j^{\prime }-0.048869)$$

j’ is defined as the day angle in radians:

(6) $$j^{\prime }=2\pi /365.25$$

### A.2. Diffuse radiation

The diffuse component *D_h_* [Wm^−2^] of the global radiation depends on the normal extraterrestrial irradiance *G*_0_, a diffuse transmission function *T_N_*, the linke turbidity factor *T_LK_* and a solar altitude function dependent *F_d_* on the solar altitude *h*_0_. For horizontal surfaces it is defined as:

(7) $$D_{h}=G_{0}T_{N}\;(T_{\mathrm{LK}})\;F_{d}\;(h_{0})$$

Calculating the diffuse radiation on inclined surfaces is more complicated. It has to be distinguished between sunlit and shadowed surfaces. Šúri and Hofierka [29] and Hofierka and Šúri [32, 39] give detailed descriptions on the equations.

## References

[1] Dorninger P., Pfeifer N. (2008). A comprehensive automated 3D approach for building extraction, reconstruction, and regularization from airborne laser scanning point clouds. Sensors.

[2] Fornather J. (2005). Schneelasten auf tragwerke neu geregelt hintergrund und auswirkung auf die baupraxis.

[3] Vögtle T., Steinle E., Tóvári D. Airborne laserscanning data for determination of suitable areas for photovoltaics.

[4] Kassner R., Koppe W., Schüttenberg T., Bareth G. Analysis of the solar potential of roofs by using official lidar data.

[5] Höfle B., Pfeifer N. (2007). Correction of laser scanning intensity data: correction of laser scanning intensity data. ISPRS J. Photogram. Remote Sens.

[6] Kraus K. (2007). Photogrammetry.

[7] Baltsavias E. (1999). Airborne laser scanning: basic relations and formulas. ISPRS J. Photogram. Remote Sens.

[8] Vosselman G. Analysis of planimetric accuracy of airborne laser scanning surveys.

[9] Oude Elberink S. Problems in automated building reconstruction based on dense airborne laser scanning data.

[10] Wehr A., Lohr U. (1999). Airborne laser scanning an introduction and overview. Int. J. Photogram. Remote Sens.

[11] Shan J., Toth C. (2008). Topographic Laser Ranging and Scanning: Principles and Processing.

[12] Rutzinger M., Höfle B., Pfeifer N., Blaschke T., Lang S., Hay G. (2008). Object detection in airborne laser scanning data - an integrative approach on object-based image and point cloud analysis. Object-Based Image Analysis - Spatial concepts for knowledge-driven remote sensing applications.

[13] Tarsha-Kurdi F., Landes T., Grussenmeyer P. Joint combination of point cloud and dsm for 3D building reconstruction using airborne laser scanner data.

[14] GRASS Development Team (2009). Geographic Resources Analysis Support System (GRASS), GNU General Public License.

[15] Matikainen L., Hyyppä J., Hyyppä H. (2003). Automatic detection of buildings from laserscanner data for map updating. Int. Archives Photogram. Remote Sens.

[16] Forlani G., Nardinocchi C. (2001). Building detection and roof extraction in laser scanning data. Int. Archives Photogram. Remote Sens.

[17] Rottensteiner F., Trinder J., Clode S., Kubik K. Automated delineation of roof planes from lidar data.

[18] Kaartinen H., Hyyppä J., Gülch E., Vosselman G., Hyyppä H., Matikainen L., Hofmann A., Mäder U., Persson A., Söderman U., Elmqvist M., Ruiz A., Dragoja M., Flamanc D., Maillet G., Kersten T., Carl J., Hau R., Wild E., Frederiksen L., Holmgaard J., Vester K. Accuracy of 3D city models: EuroSDR comparison.

[19] Tarsha-Kurdi F., Landes T., Grussenmeyer P., Koehl M. Model-driven and data-driven approaches using lidar data: analysis and comparison.

[20] Maas H.G., Vosselman G. (1999). Two algorithms for extracting building models from raw laser altimetry data. ISPRS J. Photogram. Remote Sens.

[21] Vosselman G., Dijkman S. 3D building model reconstruction from point clouds and ground plans.

[22] Wittman H., Bajons P., Doneus M., Friesinger H. (1997). Identification of roof areas suited for solar energy conversion systems. Ren. Energy.

[23] Rieger W., Seebacher M., Würländer R., Bauerhansl C. (2005). Erstellung eines laser-dhm für vorarlberg 2002 bis 2005.

[24] Pfeifer N., Stadler P., Briese C. Derivation of digital terrain models in the scop++ environment.

[25] Höfle B., Geist T., Rutzinger M., Pfeifer N. Glacier surface segmentation using airborne laser scanning point cloud and intensity data.

[26] Da T.K.F. 2D alpha shapes. CGAL-3.3 User and Reference Manual.

[27] Edelsbrunner H., Mücke E. (1994). Three-dimensional alpha shapes. ACM Trans. Graphics.

[28] Akkiraju N., Edelsbrunner H., Facello M., Fu P., Mücke E.P., Varela C. Alpha shapes: definition and software.

[29] Šúri M., Hofierka J. (2004). A new gis-based solar radiation model and its application to photovoltaic assessments. Trans. GIS.

[30] Kasten F., Young A. (1989). Revised optical air mass tables and approximation formula. Appl. Opt.

[31] Scharmer K., Greif J. (2000). The European solar radiation atlas vol. 2: Database and exploitation software.

[32] Hofierka J., Šúri M. The solar radiation model for open source gis: implementation and applications.

[33] Kasten F., Czeplak G. (1980). Solar and terrestrial radiation dependent on the amount and type of cloud. Sol. Energy.

[34] Kasten F. (1996). The linke turbidity factor based on improved values of the integral rayleigh optical thickness. Sol. Energy.

[35] NREL (2002). Nrel 2000 - solpos Documentation.

[36] Pfeifer N., Briese C. Geometrical aspects of airborne laser scanning and terrestrial laser scanning.

[37] Tanarro L., Hoelze M., Garcia A., Ramos M., Gruber S., Gomez A., Piquer M., Palacios D. (2001). Permafrost distribution modelling in the mountains of the mediterranean: Corral del veleta, sierra nevada, Spain. Norsk Geogr. Tidsskrift.

[38] Ciolli M., de Franceschi M., Rea R., Zardi D., Zatelli P. Modelling of evaporation processes over tilted slopes by means of 3d grass raster.

[39] Muneer T. (1990). Solar radiation model for Europe. Build. Serv. Eng. Res. Technol.

